# Understanding ischemia in children with tuberculous meningitis (iThemba): a protocol paper

**DOI:** 10.1186/s12887-026-06598-9

**Published:** 2026-03-13

**Authors:** Carien Truter, Melissa S. Hamilton, Camilla Le Roux, Bianca Berndorfler, Chané Buys, Magriet van Niekerk, Ronald van Toorn, Claire Dunican, Ilana van Rensburg, Kim Stanley, Sean Vermeulen, Anneke C. Hesseling, Alex Doruyter, James Warwick, Patrick Dupont, Louvina van der Laan, Christelle Ackermann, Karen du Preez, Dan Zaharie, Marlo Möller, Lindsey te Brake, Elin Svensson, Rob Aarnoutse, Myrsini Kaforou, Mike Levin, Novel Chegou, Regan Solomons, James A. Seddon

**Affiliations:** 1 Desmond Tutu TB Center, Department of Pediatrics and Child Health, Faculty of Medicine and Health Sciences, Stellenbosch University, Cape Town, South Africa; 2 Department of Infectious Disease, Imperial College London, London, UK; 3 Division of Radiodiagnosis, Department of Medical Imaging and Clinical Oncology, Faculty of Medicine and Health Sciences, Stellenbosch University, Cape Town, South Africa; 4 Division of Nuclear Medicine, Department of Medical Imaging and Clinical Oncology, Faculty of Medicine and Health Sciences, Stellenbosch University, Cape Town, South Africa; 5 Department of Pediatrics and Child Health, Faculty of Medicine and Health Sciences, Stellenbosch University, Cape Town, South Africa; 6 South African Medical Research Council Center for Tuberculosis Research, Division of Immunology, Department of Biomedical Sciences, Stellenbosch University, Cape Town, South Africa; 7 NuMeRI Node for Infection Imaging, Central Analytical Facilities, Stellenbosch University, Cape Town, South Africa; 8 Department of Neurosciences, Leuven Brain Institute, KU Leuven, Leuven, Belgium; 9 Division of Anatomical Pathology, Department of Pathology, Faculty of Medicine and Health Sciences, Stellenbosch University, Cape Town, South Africa; 10 South African Medical Research Council Center for Tuberculosis Research, Division of Molecular Biology and Human Genetics, Faculty of Medicine and Health Sciences, Stellenbosch University, Cape Town, South Africa; 11 Center for Bioinformatics and Computational Biology, Stellenbosch University, Cape Town, South Africa; 12 Genomics for Health in Africa (GHA), Africa-Europe Cluster of Research Excellence (CoRE), Stellenbosch, South Africa; 13 National Institute for Theoretical and Computational Sciences (NITheCS), Stellenbosch, South Africa; 14 Department of Pharmacy, Medical Center, Radboud University, Nijmegen, The Netherlands; 15 Department of Pharmacy, Uppsala University, Uppsala, Sweden

**Keywords:** Tuberculous meningitis, Ischemia, Penumbra, Children, Vasculitis, Infarction

## Abstract

**Background:**

One million children develop tuberculosis (TB) each year and a quarter of these die. TB meningitis (TBM) is the most severe form of TB disease and even if diagnosed and treated, 20% die and over 50% of survivors are left with permanent neurological disability. Much of the morbidity and mortality associated with TBM is due to infarction yet, despite this, our understanding of the pathogenesis of infarction in TBM remains limited, especially in children.

**Methods:**

The iThemba study (Understanding ischemia in children with tuberculous meningitis), aims to recruit 100 children with probable or confirmed TBM and obtain samples of blood and cerebrospinal fluid (CSF). All children will undergo MRI and FDG PET/CT at baseline and will have repeat MRI with further blood and CSF samples collected at 2 weeks’ follow-up. MRI will then be carried out at 24 weeks with neurodevelopmental assessment at 48 weeks. Neuroimaging will focus on methods to identify and characterize ischemic penumbra and evaluate how this correlates with clinical outcomes. RNA sequencing of blood and CSF will be used to identify differentially expressed genes and identify implicated biological pathways between children with and without infarction. Targeted proteomic profiling will be performed on plasma and CSF to determine differences in protein abundance, with a focus on proteins involved in coagulation and endothelial function. Finally, we will integrate transcriptomic, proteomic and radiomic data to generate a comprehensive understanding of the pathogenesis of infarction in children with TBM. We aim to group children into relevant biological/anatomical phenotypes, each of which may benefit from a different therapeutic approach. Using computer simulation, we will then explore the impact of potential therapeutic interventions on biological pathways for each distinct phenotype. This work may pave the way for the development of point-of-care tests at diagnosis that could allow for stratified novel therapeutic approaches in future.

**Discussion:**

A more comprehensive understanding of the pathophysiology of infarction in children with TBM would permit targeted host-directed therapies, with the potential to moderate or eliminate the consequences of this devastating condition.

## Background

Following exposure to *Mycobacterium tuberculosis* (*Mtb*) and subsequent *Mtb *infection, young children are at high risk of progression to tuberculosis (TB) disease [1, 2], and of developing disseminated forms of disease, such as tuberculous meningitis (TBM), where bacilli enter the cerebrospinal fluid (CSF), causing hydrocephalus, infarcts, and cranial nerve palsies. The peak incidence of TBM is between 2 and 4 years [3] and if untreated is fatal with the median time to death 19.5 days [4]. Of children treated, 20% die, and 50% of survivors are left with permanent neurological morbidity [5]. Even in children with good initial neurological outcomes, many have persistent learning and attention deficit later in life [6]. 

Mortality and morbidity in children with TBM are primarily driven by infarction [7], which is common, with up to half of children with TBM having infarction on magnetic resonance imaging (MRI) at the time of diagnosis [8]. Infarction is the most significant predictor of outcome [9], influenced by the number, size and location of infarcts [10]. Ischemia refers to potentially salvageable tissue with impaired perfusion/oxygenation, while infarction is dead tissue. When regional blood supply to the brain is disrupted there is commonly a core infarct, surrounded by an ischemic area, the penumbra [11]. With no intervention, this area typically progresses to infarction [12]. In adult stroke research this is a topic of intense investigation as understanding the pathophysiology is the first step to identifying therapeutic targets that could arrest ischemic progression and improve clinical outcomes.

Computed Tomography (CT) scans provide information on gross structures and pathology and can provide some information on cerebral infarct [10, 13]. Magnetic Resonance Imaging (MRI) provides the most detail for identification of both cerebral ischemia and ischemic infarct [14], with the advantage that different sequences can be used, each highlighting a specific pathological aspect. High-resolution structural imaging with good contrast between grey and white matter can be obtained using a volumetric T1-weighted structural scan such as a Magnetization Prepared Rapid Gradient Echo (MPRAGE). MRI fluid attenuated inversion recovery (FLAIR) imaging is a T2-weighted sequence with suppression of CSF and is suited to identify lacunar infarction or meningitis. MRI diffusion-weighted imaging is suitable to measure cellular oedema and therefore the presence of cerebral ischemia. MRI using arterial spin labelling (ASL) can be used to construct maps of cerebral blood flow to identify viable tissue at risk [15]. Positron Emission Tomography (PET) scanning allows for the direct visualization and quantification of biological processes such as, in the case of [18]F-fluorodeoxyglucose (FDG) PET/CT, glucose metabolism. No studies evaluating infarction in TBM to date have utilized [18]F-FDG PET/CT. Data from animal models of thromboembolic stroke show a pattern of increased glucose metabolism on FDG PET/CT in penumbral tissue in the subacute phase post-infarct [16–20], and there are some animal data to suggest that penumbral FDG is predictive of tissue fate [21, 22]. Human data includes a single study which found evidence of increased FDG uptake corresponding to ischemic lesions on MRI but did not evaluate tissue fate [23]. These data suggest a possible role for FDG to (1) identify penumbral tissue and (2) prognosticate on likelihood of tissue recovery with therapeutic intervention in infarction. Several MRI techniques for identifying penumbral tissue in thromboembolic stroke have been evaluated [24], however, none have been applied to the study of infarction in TBM. The ability of ASL MRI to measure regional cerebral blood flow, and of PET to measure regional metabolism makes these techniques well-suited to studying the perfusion-metabolism relationship in TBM ischemia. Animal studies have shown that integration of FDG PET/CT and MRI may be a sensitive approach in detecting early penumbras [22]. 

The underlying mechanisms for brain ischemia in TBM are poorly understood. A combination of thrombotic, inflammatory/vasculitic, proliferative and necrotic mechanisms have been postulated [25]. Thrombus formation in arteries and veins has been reported in several studies in adults [26], often in combination with vasculitis [27, 28]. Vasculitis is frequently found at autopsy with infiltration of leucocytes in the vessel wall, destruction of the intima and media of some vessels, and occlusive thrombi within inflamed vessels [29]. Vasculitis is also frequently detectable using cerebral imaging [9]. Veins and arteries can be affected, with a segmental or diffuse pattern [27]. Proliferation of cells in the intima and media of vessels is also commonly seen and can lead to stenosis or thickening of the intima [27]. Finally, fibrinoid necrosis can affect the intima [27, 30], the media [31] or both [32, 33], and can cause complete destruction of blood vessels.

Studies in non-TB bacterial meningitis and severe infection in children have shown that both arterial and venous thrombosis in bacterial meningitis and sepsis are associated with profound imbalance between the anticoagulant and procoagulant mechanisms on endothelium and in plasma [34, 35]. Anti-thrombotic mechanisms are down-regulated, including loss of the endothelial Protein C receptor and thrombomodulin, which together with reduced plasma levels of Antithrombin 3, Protein C and Protein S result in impaired activation of the Protein C anti-coagulant pathway [36]. Reduced endothelial production of prostacyclin [37], together with loss of endothelial glycosaminoglycans (GAGs) [38] result in platelet activation. Increased endothelial expression of tissue factor, and impaired thrombolysis due to high levels of plasminogen activator inhibitor (PAI)−1 result in a procoagulant state [39, 40]. Release of proteolytic enzymes from neutrophils and macrophages cleave endothelial surface anticoagulant GAGs, and degrade thrombomodulin and Protein C receptors, thus impairing the Protein C anticoagulant pathway [35, 41]. Recent studies in cerebral malaria and other brain inflammatory processes have shown that neutrophils release histones, resulting in NETosis [42], a process in which platelets, fibrin and other cells are trapped in occlusive thrombi [43, 44]. 

The potent angiogenic factor, vascular endothelial growth factor (VEGF), is secreted by macrophages infected by *Mtb* in pulmonary disease [45]. VEGF levels are elevated in the CSF and serum of children with TBM, compared to controls [46, 47]. In addition, VEGF production is associated with blood-brain barrier disruption [46] and ischemia [48]. Tumour necrosis factor (TNF)-α induces VEGF production [49] and may cause damage through promotion of thrombosis [50], by reduced cerebral blood flow [51], or reduction in nitric oxide production [52]. Cerebral infarction has been associated with increased CSF interleukin (IL)−8 and IL-10 in patients with TBM [53]. Altered balance of vasoactive eicosanoids synthesized by brain tissue thromboxane-2, a potent vasoconstrictor, and prostacyclin PGI2, a potent vasodilator, may lead to a dynamic ischemic injury. Alterations in procoagulant, antithrombotic, fibrinolytic, platelet and vascular endothelial functions may contribute to an increased risk of thrombosis and infarction. A decrease in anticoagulants (Protein C, S and anti-thrombin III), an increase of procoagulant factors (Factor VIII), and, raised levels of PAI-1 have been observed in children with TBM, with changes more pronounced in more advanced clinical disease state [54]. 

The overall goal of the iThemba study is to gain critical insight into the pathogenesis of brain infarction in childhood TBM and to identify distinct phenotypes at the time of diagnosis which may respond to targeted therapeutic approaches. Our long-term goal is to develop strategies, including point-of-care tests, that would allow for clinical evaluation of stratified phenotype-specific treatment and thus improve TBM outcomes in children.

## Methods

### Study design

iThemba is a prospective, observational cohort of children routinely diagnosed and treated for TBM under routine programmatic conditions, at Tygerberg Hospital in Cape Town, South Africa.

### Setting

The incidence of TB in South Africa was 427 cases per 100,000 in 2023 [55]. The City of Cape Town Metropole has a population of 4.7 million people, with 7.4 million people in the Western Cape province. Tygerberg Hospital provides tertiary pediatric services to half of this population. Each year 350–450 children are diagnosed with TB at Tygerberg Hospital [56]. Until 2016, 30–50 children were diagnosed with TBM each year. Due to Bacillus Calmette-Guérin (BCG) shortages this figure rose to 70 children with TBM in 2017 [57], an effect that has persisted, despite stock-outs being resolved. Given the success of the prevention of mother-to-child Human immunodeficiency virus (HIV) transmission program in the Western Cape with < 1% vertical transmission, 6.5% of children treated for TBM between 2017 and 2021 at Tygerberg Hospital were living with HIV [58]. The median age of children presenting with TBM at Tygerberg from 1985 to 2015 was 28 months [59]. 

### Study population

Children with symptoms and signs suggestive of TBM routinely present to Tygerberg Hospital or its drainage hospitals in the public sector, where all people with TB are routinely treated free of charge. Symptoms and signs of TBM include ≥1 of headache, irritability, vomiting, fever, neck stiffness, convulsions, focal neurological deficits, altered consciousness, or lethargy [60]. Local standard of care includes referral for emergency CT scan followed by lumbar puncture (LP) to obtain samples and complete an air encephalogram. If the history, examination, and investigations suggest TBM, the child is started on TBM therapy.

For the study, all children suspected of having TBM who are routinely admitted to Tygerberg Hospital will be evaluated for study eligibility and recruited prior to initiation of TBM therapy. We will enrol 100 children with probable or definite TBM using standard diagnostic criteria [60], with recruitment over approximately 48 months. We will recruit all children evaluated for TBM and will carry out CT imaging and CSF and blood draws in all at baseline, since at the time of initial evaluation, the diagnosis of TBM may not yet be determined. FDG PET/CT, MRI and repeat sampling will only be completed in children with a diagnosis of TBM (probable or definite TBM). Given the low prevalence of HIV in children with TBM in the study setting, it is likely that in our prospective cohort fewer than 5 children with TBM will have HIV. Given the potential for analytic bias and the limited information available from such a small subgroup, we will include children with TBM living with HIV for descriptive purposes only. The main analysis will be restricted to children without HIV.

### Clinical management

Local guidelines recommended treating TBM with a short, intensive 4-drug regimen consisting of daily isoniazid 20 mg/kg (maximum 450 mg daily), rifampicin 30 mg/kg (maximum 900 mg daily), pyrazinamide 45 mg/kg (maximum 2 g daily) and ethionamide 22.5 mg/kg (maximum 1 g daily), all given once-daily for 6 months. HIV-positive children are treated for 9 months for TBM. Prednisone 2 mg/kg/day (maximum 60 mg/day) is given for the first month of treatment and gradually discontinued over the next 2 weeks. If the child’s *Mtb* isolate or that of the index patient is resistant to any of the drugs used or if there is clinical deterioration, individualized TB treatment is given based on the drug susceptibility pattern of the index patient or the child. Air-encephalography is used to distinguish between communicating and non-communicating hydrocephalus. Hyponatremia is corrected with slow administration of intravenous hypertonic saline.

### Clinical procedures and follow up

Standard clinical data will be collected in a RedCap database as per local standard of care (Table 1) including TBM stage, sociodemographic data, other medical conditions, previous TB treatment, HIV exposure and infection status, vaccination history (including BCG and other routine vaccinations), anthropometrics, and details of the illness leading to the current admission [61]. All children will have a CXR (antero-posterior and lateral films), and a minimum of two respiratory samples (expectorated sputum, induced sputum or gastric aspirate) collected for Xpert MTB/RIF Ultra (Cepheid, Sunnyvale, CA, USA) and mycobacterial culture. All children will be tested for HIV (ELISA if ≥ 18 months and PCR if < 18 months or if breast-feeding is ongoing); if positive, CD4 and viral load will be measured, and the child immediately initiated on combination antiretroviral therapy (cART). The routine attending clinical care team will make the decision to start TBM treatment. All children treated for TBM are routinely seen every 4 weeks while on treatment. In addition, the study team will evaluate children at weeks 1, 2, 8, 24, 48, 78 and 104 with formal neurodevelopmental assessment at 48, 78 and 104 weeks, undertaken by a pediatrician using the Griffiths III neurodevelopmental scales [62, 63]. A Strengths and Difficulties Questionnaire (SDQ) and a Developmental and Well-Being Assessment (DAWBA) will be performed at week 48, 78 and 104 to assess for neurobehavioral sequelae post-TBM [64–66]. 

**Table 1:** Schedule of investigations in children with TB meningitis in the iThemba study

	Baseline	Week 1	Week 2	Week 8	Week 24	Week 48	Week 78	Week 104
History and clinical review	●	●	●	●	●	●	●	●
HIV status (HIV Elisa or HIV PCR)	●							
Chest radiograph	●							
Two respiratory samples^1^	●							
Neurodevelopmental assessments						●	●	●
Health-related Quality of Life questionnaire				●	●	●	●	●
Socio-economic questionnaire				●	●	●	●	●
Neurobehavioral assessments						●	●	●
Research blood samples^2^	●		●	●	●			
Lumbar puncture	●		●					
Computed tomography of the head	●							
Non-invasive optic pressure measurement	●		●					
Magnetic resonance imaging brain	●		●		●			
^18^F-FDG-PET/CT	●							

*HIV* Human Immunodeficiency Virus and *F-18 FDG PET/CT* fluorine-18 fluorodeoxyglucose positron emission tomography/computed tomography

All children will undergo LP at baseline and, for those with TBM, at 2 weeks. Opening pressures are routinely measured in all children. Non-invasive measuring of intracranial pressure, with a transcranial doppler or optic pressure tool, will be done during lumbar punctures. Blood sampling will be done at weeks 2, 8 and 24. All children will have urgent CT scan performed as per routine care to support the diagnosis and guide immediate treatment. Study participants with TBM will have an MRI scan and an FDG PET/CT scan in the subsequent week with a repeat MRI at 2 weeks and at 24 weeks, or in addition as clinically indicated.

MRI scans will be performed on a Siemens MAGNETOM Aera 1.5T. The following sequences will be acquired: a structural T1 weighted scan (T1 MPRAGE), T2 FLAIR, diffusion weighted imaging (at b = 0 and b = 1000 s/mm2), T2 susceptibility weighted imaging SWI, and a perfusion MRI using ASL. From the ASL measurements, a cerebral blood flow (CBF) map will be calculated using the scanner software and from the diffusion weighted imaging, an apparent diffusion coefficient (ADC) map will be calculated. FDG PET-CT scans will be performed on a Philips Vereos PET-CT camera after injection of a weight-adjusted dose of FDG calculated according to the European Association of Nuclear Medicine (EANM) dosage recommendations. Brain PET-CT imaging will be performed after an uptake time of 45 min and will be acquired over 10 min (5 × 2 min frames) [67]. A second, whole-body acquisition will be commenced immediately after. Both brain and whole-body PET acquisitions will be performed in combination with low-dose CT with dose-sparing measures. An experienced pediatrician will manage participant sedation for the imaging exams, as necessary.

### Study aims

**Fig. 1:**
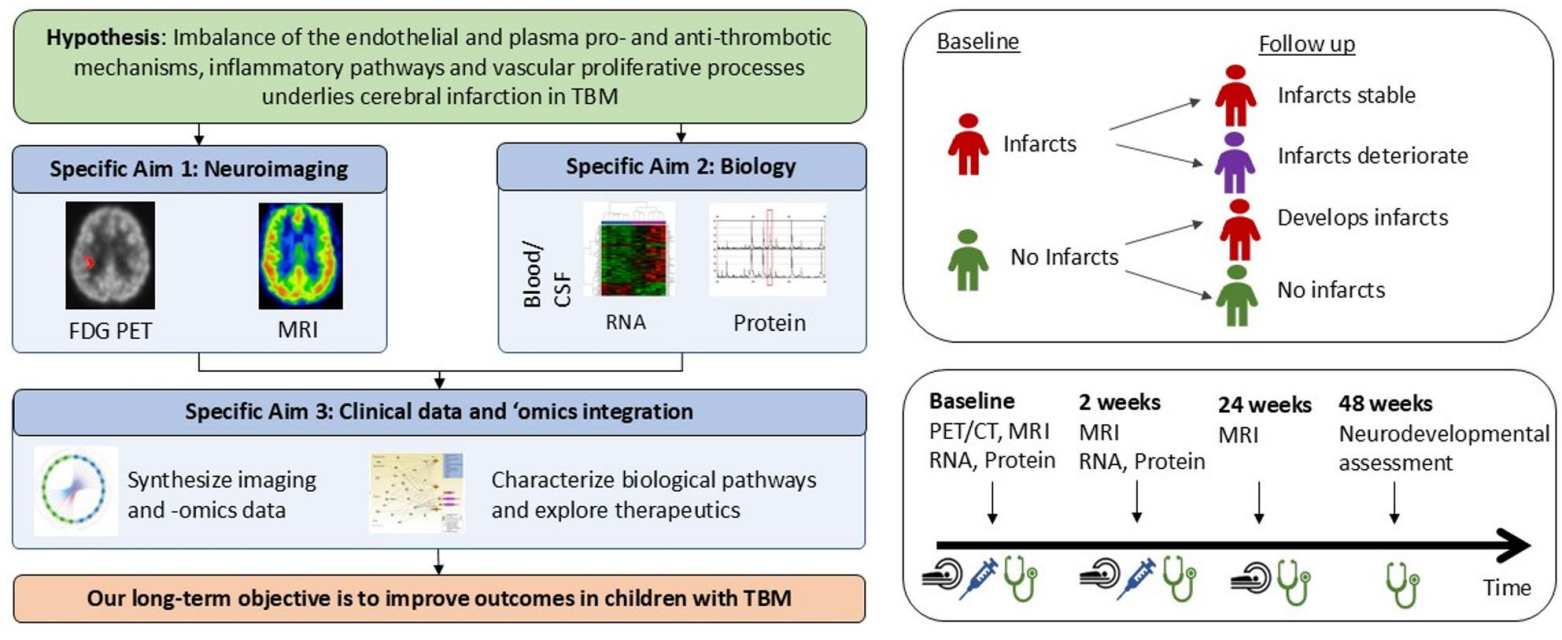
Study Overview of children with tuberculous meningitis in the iThemba study

We will investigate the following aims (Fig. 1):

1. To identify features on neuroimaging at baseline that predict the development/evolution of ischemic infarcts and long-term neurodevelopmental status in children with TBM.
2. To identify the pathogenic mechanisms in TBM that distinguish children with and without cerebral infarction seen on neuroimaging.
3. To integrate radiomic, transcriptomic and proteomic data with clinical parameters at baseline and at follow up to characterize the biology of infarction in childhood TBM.

### Aim 1. To identify features on neuroimaging at baseline that predict the development/evolution of ischemic infarcts and long-term neurodevelopmental status in children with TBM

#### Overview

We will investigate the hypothesis that penumbral areas can be detected at baseline, and that they can predict future infarction. Specifically, we will use [18]F-FDG PET/CT and multiple MRI sequences to measure the size and characteristics of infarct and penumbra at sequential timepoints.

#### Analysis

**Fig. 2:**
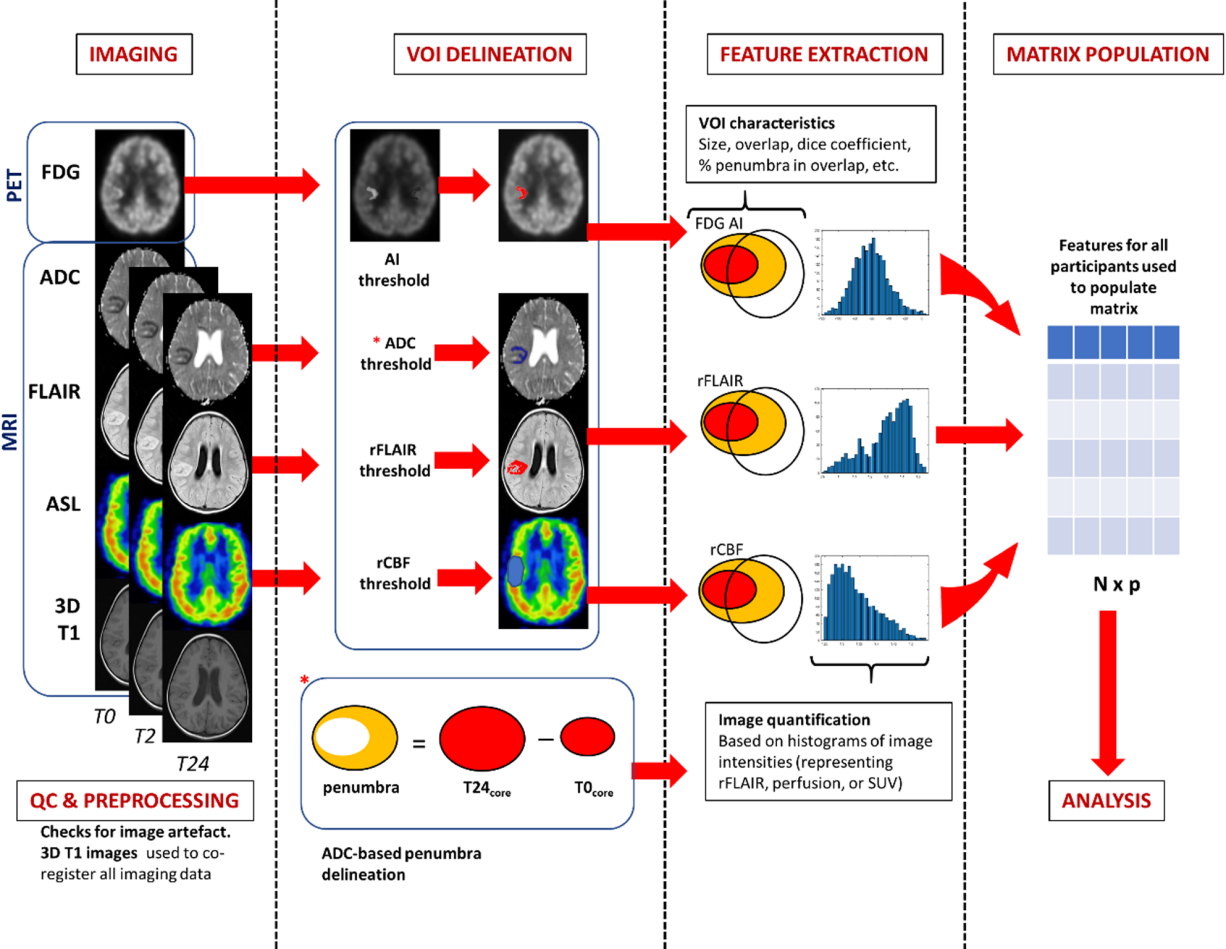
Proposed imaging pipeline. Penumbral tissue (orange) is defined as ischemic core (red) at baseline or two weeks subtracted from final ischemic core at 6 months Abbreviations: PET: positron-emission tomography, FDG:[fluorine-18] fluorodeoxyglucose; MRI: magnetic resonance imaging; ADC: apparent diffusioncoefficient; (r)FLAIR: (relative) fluid-attenuated inversion recovery; ASL: arterial spin labelling;3D T1: 3-dimensional T-1 weighted image; T0: baseline; T2: 2-week; T24: 6 month; QC: qualitycontrol; VOI: volume of interest; AI: asymmetry index; rCBF: regional cerebral blood flow; SUV:standard uptake value

Final core (infarct) will be defined on ADC images at 24 weeks. Penumbra will be defined as ischemic core at either baseline (for ischemic lesions present at baseline) or two-weeks (for ischemic lesions that only manifest on 2-week scan) subtracted from the final core (Fig. 2). Utilizing these reference segmentations as masks, we will evaluate how other neuroimaging delineations of penumbra on different sequences (volumes of interest [VOIs]) calculated according to e.g., asymmetry index on FDG, ADC-threshold, FLAIR-threshold, regional cerebral blood flow threshold on ADC maps) compare (volume, overlap, dice-coefficients, etc.), and how these predict final core burden (on ADC map) and clinical outcome at 24-weeks. These measures, as well as first- (± higher-) order radiomic measures calculated from the different penumbral VOI histograms of voxel intensities, will be fed into multi-omics analysis. We will use the multiple imaging parameters to check for multi-collinearity and will then select a subset of demographic and clinical variables as explanatory variables, including age, gender, TBM stage and nutritional status. We will use a backward regression approach to determine the final model, using Akaike information criteria. Prediction models making use of deep learning have been successfully applied in other research contexts and will also be explored [68–71]. 

### Aim 2. To identify the pathogenic mechanisms in TBM that distinguish children with and without cerebral infarction observed on neuroimaging

#### Overview

We will investigate the hypothesis that imbalance of the endothelial and plasma pro- and anti-thrombotic mechanisms, inflammatory pathways and vascular proliferative processes underlies cerebral infarction in TBM, as it does in other severe infections [34, 72, 73]. 

#### RNA expression profiling of blood and CSF

##### RNA extraction and sequencing

Blood will be collected in PAXgene Blood ribonucleic acid (RNA) Tubes (Qiagen), and total RNA will be extracted using the PAXgene Blood RNA Kit (Qiagen). RNA extractions will be measured for quantity, quality and purity then stored at −80 °C for transfer to the sequencing facility. CSF will be collected in universal containers but immediately spun and the pellet dissolved in Trizol and frozen at −80 °C. RNA will be extracted using Direct-zol RNA Microprep Kit (Zymo). RNA-sequencing (RNA-Seq) for both blood and CSF will be done at the South African Medical Research Council Genomics Centre and sequenced at PE150 on an MGI-SEQ2000 machine with > 50 reads/sample. Previous work has demonstrated excellent read quality [74]. 

##### RNA-sequencing analysis

The bioinformatic pipeline will include: (1) Sequencing quality control to assess for genomic contamination and globin/ribosomal RNA depletion, (2) RNA-Seq data quality control using FastQC [75], MultiQC [76] and annotations modified with BEDTools [77], (3) alignment and read counting using STAR [78], SAMtools [79], FeatureCounts [80] and version 89 ensembl GCh38 genome [81] and (4) annotation. Gene counts will be imported into R [82] for normalization using DESeq2 [83]. Differences in gene expression relating to infarction will be quantified as log2 of the fold change using DESeq2 accompanied by correction for multiple testing and with correction for relevant clinical covariates, such as age and sex. The analysis for the blood RNA-Seq data will be performed with and without correction for the relative proportions of different peripheral blood cell types [84]. We will use gene ontology enrichment (GO) analysis and Ingenuity Pathway Analysis (IPA) software (Qiagen) to pinpoint biological pathways [85]. A similar approach will be used to relate differential gene expression to neurodevelopmental outcome at 48 weeks, using both a dichotomous Griffiths score above and below 70, as well as a continuous variable.

#### Proteomic profiling of plasma and CSF

CSF supernatant and plasma will be analysed on the SomaScan platform, which utilises the SOMAmer (Slow Off-rate Modified Aptamer) reagents consisting of short single-stranded deoxyribonucleic acid (DNA) sequences that allow tight and specific binding of proteins to their target. This specificity has enabled the current SomaScan platform (v 4.1) to accurately resolve over 11,000 proteins. Samples will be analysed in a single run and at three plasma concentrations (20%, 0.5%, 0.005%) and three CSF concentrations (10%, 0.5%, 0.05%) to ensure the precise measurement of low, medium and highly abundant proteins and the raw fluorescence data returned for analysis. Data validation and quality control steps will be performed using internal assay controls to correct for variations in aptamer hybridisation efficiency, inter- and intra-assay variability. We will use differential abundance analysis (DAA) to identify proteins that are significantly different between children with and without infarction. IPA will be used to identify biological pathways involved in the pathogenic mechanisms in TBM that distinguish children with and without cerebral infarction.

### Aim 3. To integrate radiomic, transcriptomic and proteomic data with clinical parameters at baseline and at follow up to characterize the biology of infarction in children with TBM

#### Multi-omics integration approach

Various methods of integration have been developed, including graph-based methods [86, 87], clustering-based methods [88, 89], and factor analysis-based methods [90–92]. We will perform supervised and unsupervised integration analyses using these methods to identify the mechanisms that distinguish children with and without infarct. First, the acquired RNA and protein profiling data, along with the clinical and the FDG PET/CT and MRI data will be pre-processed and analysed on a single -omics level. Count data from the RNA dataset, abundance levels from the protein dataset, a set of features summarizing the FDG PET/CT and MRI data, along with clinical data, will be used as an input for the integration across all patients. First, we will aim to identify a multi-omic set of features capable of distinguishing children with and without infarction. We will use DIABLO [93] and in addition will perform a data-driven hypothesis free analysis to identify clusters of patients with similar -omics profiles. To this end we will use Multi-Omics Factor Analysis (MOFA) which is a versatile generalization of PCA to multi-omics data [90] as it infers a low-dimensional representation of the data. MOFA allows missing values to be imputed, and factors to be annotated through gene set enrichment analysis. The molecular features identified by MOFA from the inferred factor loadings could be used as a complement to the biomarkers identified by DIABLO [90]. In addition to MOFA we will use similarity network fusion (SNF) [86] for unsupervised analysis and disease subtype identification. SNF creates a matrix for each ’omic type (RNA, protein, PET/CT, MRI and clinical) to identify pairwise sample similarities [86] and constructs a network for each data type. Finally, a single consensus network will be produced which contains shared and complementary information from the data sources [86]. From this, clusters of patients will be identified with greater resolution than the single ‘omics networks allow. We aim to identify several groupings, or phenotypes, of children that have similar mechanisms of infarction and who might benefit from similar therapeutic approaches, including host-directed therapies. Supervised and unsupervised integration analyses will be repeated to identify the mechanisms that distinguish children identified in Aim 1 who have infarct and penumbra versus children with infarct and no penumbra. Finally, we will repeat supervised and unsupervised integration analyses to determine factors influencing clinically assessed neurodevelopmental status at 48 weeks. This will be conducted to discriminate children with a Griffiths score above and below 70, as well using the neurodevelopmental score as a continuous variable.

#### Evaluation of potential therapeutic interventions in Silico

After identifying clinically relevant patient phenotypes, we will assess the in-silico effect that different drugs could potentially have on pathways that are most de-regulated in each subtype by using an in-house computational network biology approach. Using differential expression/abundance analysis, we will map the expression levels of genes and proteins on the different pathways. To account for the effects of crosstalk signalling between pathways, we will employ a method from cancer medicine, with cross-talk referring to protein interactions shared between distinct pathways [94]. The therapeutic networks associated with each drug will be compiled using IPA, the Kyoto Encyclopaedia of Gene and Genomes (KEGG) and the Reactome databases. Directed networks including proteins will be constructed and their interactions will be used, where the type of an interaction, such as activation or inhibition, will be reflected with the directionality of an edge in the network. A crosstalk inhibition/activation measure will be used to estimate the amount of crosstalk signalling that can be prevented/enhanced between pathways by inhibiting/activating specific proteins simultaneously. Using topology-based measures of network efficiency, we will compute the flow of information within a crosstalk network before and after inhibiting individual or combined drug targets. Drug combinations with high impact are expected to present promising candidates for further future exploration including in controlled clinical trials. These are likely to include anti-inflammatory, anticoagulant, antithrombotic, anti-platelet, or thrombolytic agents (Table 2). We will then also assess the effects of drugs or combinations of drugs on key molecules of the deregulated pathways by using the Molecule Activity Predictor functionality in IPA which interrogates single pathways simulating directional consequences of downstream molecules and the inferred activity upstream in the network or pathway, while perturbing the expression levels of key molecules in silico.

**Table 2:** Potential future therapeutic agents for consideration based on mechanism of vascular occlusion in children with TB meningitis

Mediator of Vascular Occlusion	Potential Therapeutic Agent
Neutrophil	Colchicine
Neutrophil-derived proteases and NETs	Protease inhibitors, glycosaminoglycans
Macrophages	Steroids
T-cells	Cyclosporin, calcineurin inhibitors
Endothelial cell dysfunction	Prostacyclin, aspirin
Coagulation	Anti-coagulants
Defective anti-coagulation	Activated Protein C
Impaired thrombolysis	tPA, streptokinase
Platelet	Prostacyclin, clopidogrel, aspirin, 2b3a inhibitor

## Discussion

Our overall goal is to improve outcomes for children with TBM, the most severe form of pediatric TB despite effective antituberculosis drugs being available. Infarct is the leading cause of morbidity and mortality in children with TBM despite appropriate chemotherapeutic antimycobacterial treatment, yet our current understanding of its pathophysiology remains poor. We plan to use state-of-the-art complementary approaches to investigate a well-characterized cohort of children with TBM with well-defined neurodevelopmental outcomes to gain insight into the pathogenesis of ischemic TBM. We will identify children with different biological phenotypes of ischemia and then evaluate, in silico, different drugs and drug combinations as potential therapeutic interventions. We anticipate that the results of this study will lead to the prospective controlled clinical evaluation of novel host-directed and other therapeutic approaches in children with TBM. Finally, the results of our study will inform the development of point-of-care tests to discriminate different patient phenotypes and controlled trials of targeted therapeutic interventions. This work in childhood TBM may also have relevance for the treatment of adult TBM in future.

### Sample size and current status

We aim to recruit 100 children with probable or definite TBM. We based our sample size calculation based on the RNA sequencing analysis. In a pilot RNA-seq data analysis, the total number of genes tested was 20,000, the top 250 genes were significantly differentially expressed (SDE), with a minimum average read counts of 30, and a maximum dispersion of 0.4. If the desired minimum fold change is 1.6, (which was the case for 120 SDE genes in the pilot study), we will be able to reject the null hypothesis that the population means of the two groups are equal with probability (power) 0.90. The false discovery rate (FDR) is 0.05. Recruitment started on 19 June 2023. On 04 June 2025 we recruited 56 participants with TBM. Recruitment is expected to continue through to May 2027.

### Sub-studies

The primary question addressed within this study is an analysis of the biological underpinning of ischemia in children with TBM. However, this study provides a platform to carry out multiple other investigations. These include pharmacokinetic and pharmacogenomic analyses, (using a limited pharmacokinetic sampling scheme, with sampling occurring at multiple occasions), TBM genetic susceptibility investigations, diagnostic work, socio-behavioural research, health systems and health economic evaluations. We will collect appropriate samples and clinical data to enable investigation of these other important research questions as sub-studies.

## Data Availability

De-identified data will be made available on an open access platform upon completion of the study.

## Abbreviations

Mtb: Mycobacterium tuberculosis
TB: Tuberculosis
TBM: Tuberculous meningitis
CSF: Cerebrospinal fluid
MRI: Magnetic Resonance Imaging
CT: Computed Tomography
MPRAGE: Magnetization-Prepared Rapid Acquisition Gradient Echo
FLAIR: Fluid-Attenuated Inversion Recovery
ASL: Arterial Spin Labelling
PET: Positron Emission Tomography
FDG: Fluorodeoxyglucose
GAGs: Glycosaminoglycans
PAI: Plasminogen activator inhibitor
VEGF: Vascular endothelial growth factor
TNF: Tumour necrosis factor
IL: Interleukin
BCG: Bacillus Calmette-Guérin
HIV: Human immunodeficiency virus
LP: Lumbar puncture
CXR: Chest x-ray
ELISA: Enzyme-linked immunosorbent assay
PCR: Polymerase Chain Reaction
CD4: Cluster of differentiation 4
cART: Combination antiretroviral therapy
SDQ: Strengths and Difficulties Questionnaire
DAWBA: Developmental and Well-Being Assessment
CBF: Cerebral Blood Flow
ADC: Apparent Diffusion Coefficient
T1/T2: Longitudinal Relaxation Time
EANM: European Association of Nuclear Medicine
VOI: Volumes of interest
RNA: Ribonucleic acid
RNA-seq: RNA-sequencing
GO: Gene Ontology
IPA: Ingenuity Pathway Analysis
SOMAmer: Slow Off-rate Modified Aptamer
DNA: Deoxyribonucleic acid
DAA: Differential abundance analysis
MOFA: Multi-Omics Factor Analysis
SNF: Similarity network fusion
IPA: Interpretative Phenomenological Analysis
KEGG: Kyoto Encyclopaedia of Gene and Genomes
SDE: Significantly differentially expressed
FDR: False discovery rate
